# TurboID Screening of the OmpP2 Protein Reveals Host Proteins Involved in Recognition and Phagocytosis of *Glaesserella parasuis* by iPAM Cells

**DOI:** 10.1128/spectrum.02307-22

**Published:** 2022-09-12

**Authors:** Changsheng Jiang, Ningning Ma, Hua Cao, Wei Zeng, Jingping Ren, Yaofang Hu, Jiaru Zhou, Mengjia Zhang, Chang Li, Yifei Lang, Wentao Li, Qigai He

**Affiliations:** a State Key Laboratory of Agricultural Microbiology, College of Animal Sciences and Veterinary Medicine, Huazhong Agricultural University, Wuhan, China; b The Cooperative Innovation Center for Sustainable Pig Production, Wuhan, China; c Key Laboratory of Prevention and Control Agents for Animal Bacteriosis, Ministry of Agriculture and Rural Affairs, Institute of Animal Husbandry and Veterinary, Hubei Academy of Agricultural Sciences, Wuhan, China; d Swine Disease Research Center, College of Veterinary Medicine, Sichuan Agricultural University, Chengdu, China; e Hubei Hongshan Laboratory, Wuhan, China; Griffith University

**Keywords:** *Glaesserella parasuis*, outer membrane protein P2 (OmpP2), proximity labeling, TurboID, interacting proteomes

## Abstract

Glaesserella parasuis is a common bacterium in the porcine upper respiratory tract that causes severe Glasser’s disease, which is characterized by polyarthritis, meningitis, and fibrinous polyserositis. TurboID is an enzyme that mediates the biotinylation of endogenous proteins that can fuse with proteins of interest to label protein interactors and local proteomes. To reveal the host proteins that interact with outer membrane protein P2 (OmpP2) by TurboID-mediated proximity labeling in immortalized porcine alveolar macrophage iPAM cells, 0.1 and 2.58 mg/mL His-tagged TurboID-OmpP2 and TurboID recombinant proteins were expressed and purified. By mass spectrometry, we identified 948 and 758 iPAM cell proteins that interacted with His-TurboID-OmpP2 and His-TurboID, respectively. After removal of background proteins through comparison with the TurboID-treated group, 240 unique interacting proteins were identified in the TurboID-OmpP2-treated group. Ultimately, only four membrane proteins were identified, CAV1, ARF6, PPP2R1A, and AP2M1, from these 240 host proteins. Our data indicated that CAV1, ARF6, and PPP2R1A could interact with OmpP2 of G. parasuis, as confirmed by coimmunoprecipitation assay. Finally, we found that CAV1, ARF6, and PPP2R1A were involved in the recognition and phagocytosis of *G. parasuis* serotype 5 by iPAM cells by using overexpression and RNA interference assays. This study provides first-hand information regarding the interaction of the iPAM cell proteomes with *G. parasuis* OmpP2 protein by using the TurboID proximity labeling system and identifies three novel host membrane proteins involved in the recognition and phagocytosis of *G. parasuis* by iPAM cells. These results provide new insight for a better understanding of Glasser’s disease pathogenesis.

**IMPORTANCE** *G. parasuis* can cause serious Glasser’s disease, which is characterized by polyarthritis, meningitis, and fibrinous polyserositis in pigs. It can cause high morbidity and mortality in swine herds and major economic losses to the global pig industry. Understanding the mechanism of interactions between alveolar macrophages and pathogenic *G. parasuis* is essential for developing effective vaccines and targeted drugs against *G. parasuis*. To reveal the host proteins interacting with OmpP2 by TurboID-mediated proximity labeling in immortalized porcine alveolar macrophage (iPAM) cells, we identified 240 unique proteins from iPAM cells that could interact with *G. parasuis* OmpP2. Among them, only four membrane proteins, CAV1, ARF6, PPP2R1A, and AP2M1, were identified, and further study showed that CAV1, ARF6, and PPP2R1A are involved in the recognition and phagocytosis of *G. parasuis* serotype 5 by iPAM cells. This study provides new insight into proteomic interactions between hosts and pathogenic microorganisms.

## INTRODUCTION

Glaesserella parasuis is a Gram-negative bacterium and an important member of the *Pasteurellaceae* family. Its growth strictly depends on the V factor (nicotinamide adenine dinucleotide [NAD]) (1–3). G. parasuis can cause Glasser’s disease, a serious disease characterized by polyarthritis, meningitis, and fibrinous polyserositis in pigs under specific circumstances, such as stress and immunosuppression (4, 5). At present, at least 15 serotypes of *G. parasuis* have been identified. In China, the most prevalent serotypes are serotypes 4 and 5, followed by serotypes 13, 14, and 12, and approximately 12% of strains isolated from farms are nontypeable (6, 7). Outer membrane protein P2 (OmpP2) of *G. parasuis*, a member of the porin family, is one of the most abundant proteins located on the outer membrane and features eight surface-exposed loops (8, 9). OmpP2 plays an essential role in maintaining the permeability and integrity of the membrane and mediating interactions with host cell proteins associated with pathogenesis by Gram-negative bacteria (9–11). A previous study found that OmpP2 was involved in the adhesion and invasion of porcine umbilical vein endothelial cells (PUVECs) and porcine kidney epithelial cells (PK-15), suggesting that OmpP2 is a virulence factor of *G. parasuis* (12). However, the mechanisms of OmpP2 involved in *G. parasuis* pathogenesis are still unclear. Therefore, this study aimed to reveal the host proteins interacting with OmpP2 by TurboID-mediated proximity labeling in immortalized porcine alveolar macrophage (iPAM) cells and the recognition and phagocytosis mechanisms of *G. parasuis* by iPAM cells.

In recent years, proximity labeling methods to identify protein-protein interactions have attracted researchers’ attention. Tools such as APEX (13, 14), antibody-based approaches such as EMARS (15), and biotin-ligase-based approaches such as BirA^R118G^ (BioID) (16, 17) were developed to study protein-protein interactions. However, those methods also have limitations that restrict their applications. For instance, the APEX system offers rapid peroxidase-based labeling, but the utilization of H_2_O_2_ is challenging due to its toxicity to cells. In addition, the accuracy of the EMARS method is mainly dependent on the quality of the antibody used. BioID-based methods require a relatively long labeling time (approximately 15 h) to label interacting proteins. Recently, a new labeling enzyme called TurboID, a mutant of the Escherichia coli biotin ligase BirA with faster labeling kinetics, was developed, and it requires only 10 min to label proteins of interest (18). TurboID is an enzyme that can catalyze the biotinylation of endogenous proteins, which can then fuse to a protein of interest to label interacting proteins or protein complexes. When free biotin is supplied in the presence of ATP, TurboID binds and activates biotin and then releases reactive biotinyl-AMP, which can covalently bind to nearby primary amines on lysine residues that occur within an ≈10-nm radius (17, 19, 20). Biotinylated proteins can be captured by high-affinity streptavidin magnetic beads and characterized by mass spectrometry (11). Previous studies demonstrated that TurboID is a powerful tool for studying protein-protein interactions both *in vitro* and *in vivo* (11, 18). To the best of our knowledge, there has been no report of applying TurboID to study bacteria-host interactions.

In the present study, we applied a TurboID proximity labeling method to identify iPAM cell proteins that interacted with *G. parasuis*. His-tagged TurboID-OmpP2 and TurboID recombinant proteins were expressed and purified. OmpP2-specific interacting proteins were identified by mass spectrometry. The membrane proteins were identified using the UniProt website, and their interaction with OmpP2 was confirmed by coimmunoprecipitation (co-IP) assay. Finally, through overexpression and RNA interference assays, we provide evidence of the roles of CAV1 (caveolin 1), ARF6 (ADP ribosylation factor 6), and PPP2R1A (protein phosphatase 2 scaffold subunit Aα) in the recognition and phagocytosis of *G. parasuis* serotype 5 by iPAM cells.

## RESULTS

### Expression of His-TurboID-OmpP2 and His-TurboID and validation of their biotinylation efficiencies.

**FIG 1:**
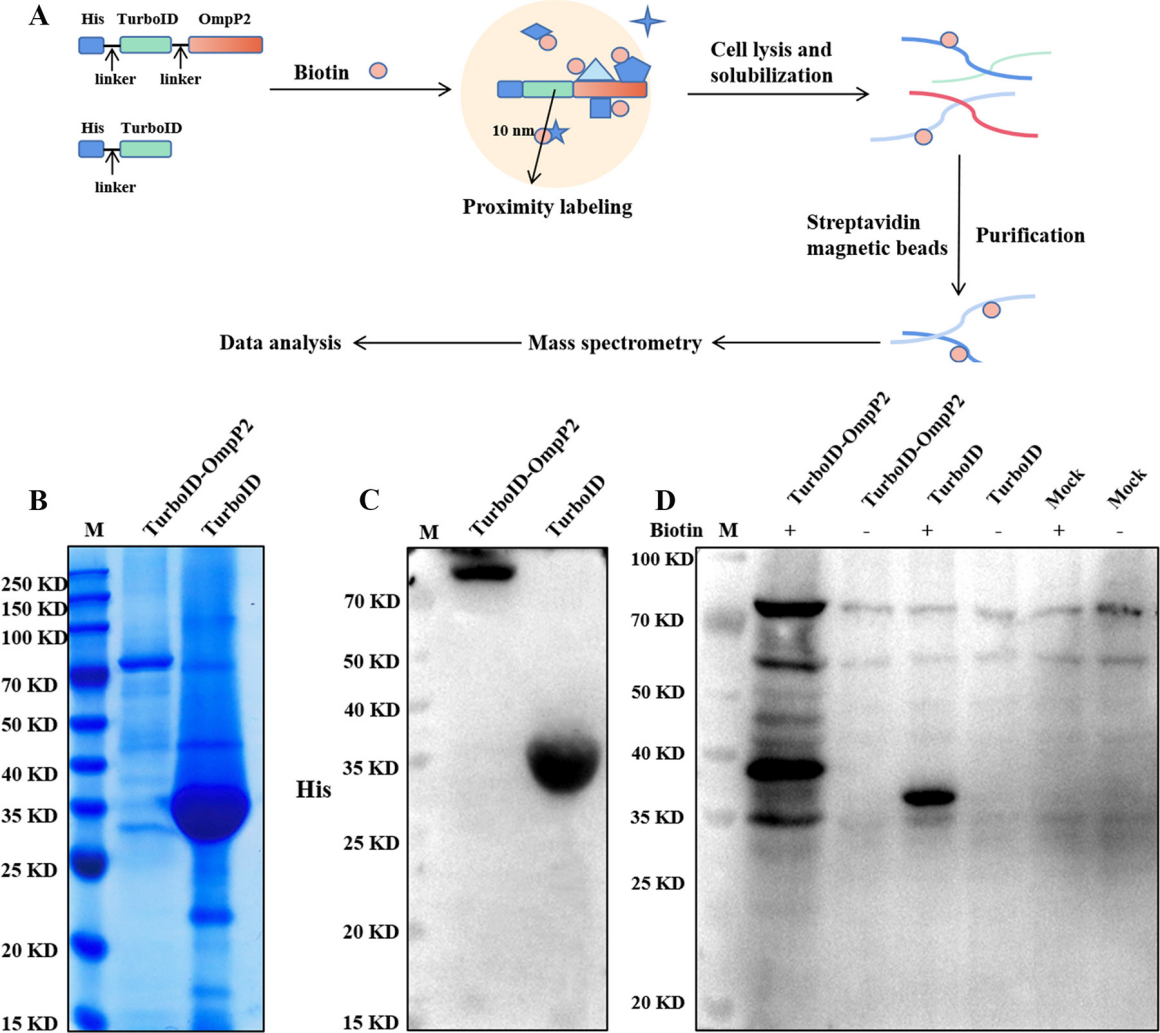
Expression of His-TurboID-OmpP2 and His-TurboID and validation of their biotinylation efficiencies. (A) Schematic overview of the TurboID-mediated proximity biotinylation assay using His-TurboID-OmpP2 and His-TurboID. (B) Analysis of His-TurboID-OmpP2 and His-TurboID recombinant protein expression by SDS-PAGE. (C) Analysis of His-TurboID-OmpP2 and His-TurboID recombinant protein expression by Western blotting. (D) Western blot analysis of His-TurboID-OmpP2-treated and His-TurboID-treated iPAM cells. iPAM cells were treated with His-TurboID-OmpP2 or His-TurboID or were not treated in medium with and without supplementation with 50 μM biotin. Total cell lysates were detected by Western blotting using a probe with HRP-coupled streptavidin.

To produce a fusion protein for proximal biotin labeling, the OmpP2 gene of *G. parasuis* serotype 5 was fused to the TurboID gene with a 6-amino-acid linker (GGSGGS) in the expression vector pET-28a and a His tag fused to the 5′ end of TurboID (Fig. 1A). The expression plasmid His-TurboID served as a control. The expression plasmids His-TurboID-OmpP2 and His-TurboID were transformed into E. coli BL21(DE3), and the corresponding fusion proteins His-TurboID-OmpP2 and His-TurboID were detected by SDS-PAGE and Western blotting. The recombinant His-TurboID-OmpP2 and His-TurboID were confirmed by SDS-PAGE and Western blotting (Fig. 1B and C).

To further evaluate the efficiency and specificity of TurboID-mediated biotinylation, we analyzed by Western blotting the fractions of biotinylated proteins derived from His-TurboID-OmpP2, His-TurboID, or mock-treated iPAM cells with or without free biotin in the culture medium. Similar patterns of endogenous biotinylated proteins were observed in all groups when no exogenous biotin was added to the culture medium. In addition, a similar biotinylated protein pattern was observed in mock-treated iPAM cells in culture medium with or without 50 μM biotin, indicating that the biotinylation of endogenous proteins depended on TurboID expression. In contrast, we observed a significantly increased fraction of biotinylated proteins in the lysates derived from iPAM cells treated with His-TurboID-OmpP2 or His-TurboID in the presence of free biotin. In addition, the fraction of biotinylated proteins in His-TurboID-OmpP2 fusion protein-treated cells was much higher than that in His-TurboID fusion protein-treated cells (Fig. 1D). These results showed that both His-TurboID-OmpP2 and His-TurboID could efficiently biotinylate target proteins in the presence of free biotin in the culture medium.

### Determination of the *G. parasuis* OmpP2-proximal proteome.

**FIG 2:**
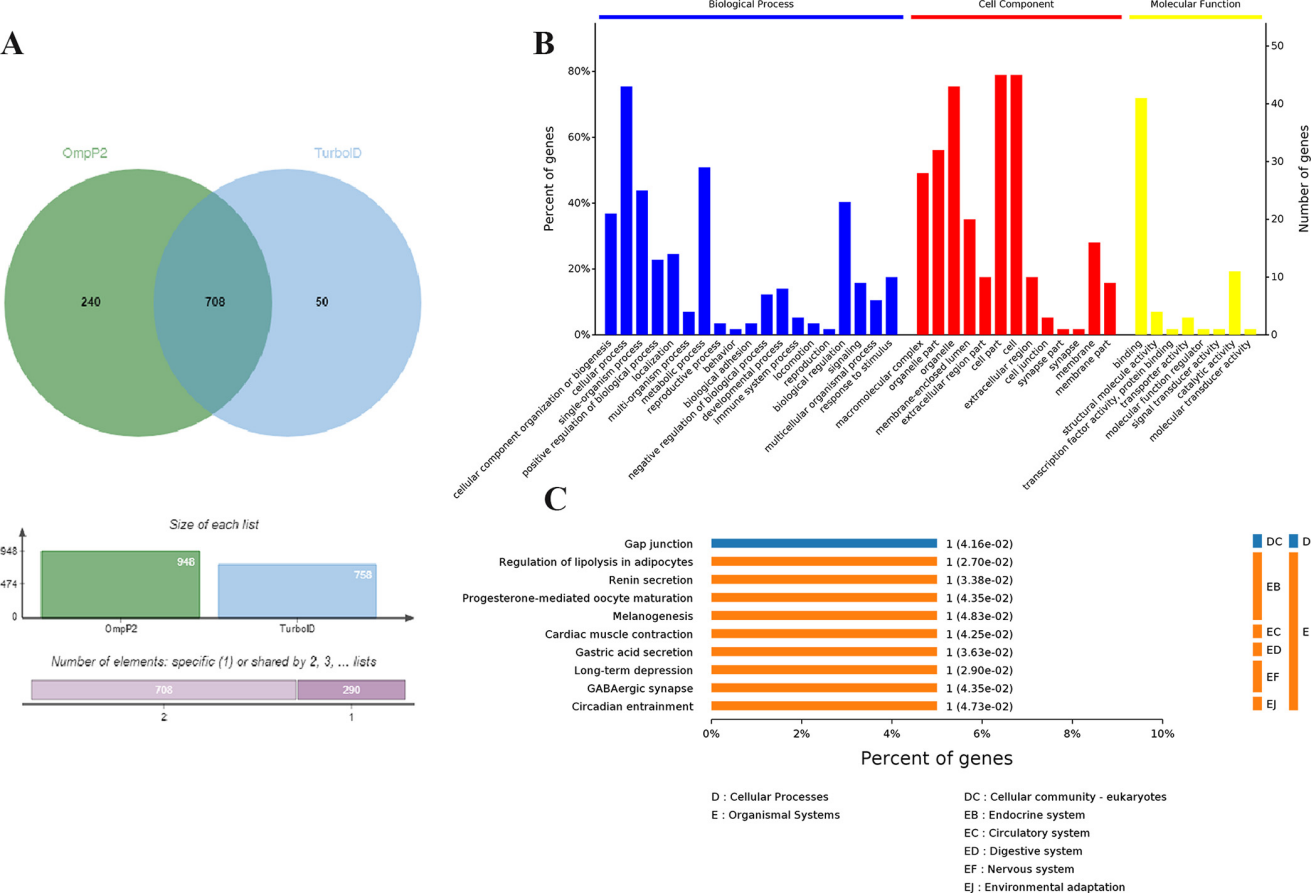
Bioinformatics analysis of host cell proteins putatively identified as interacting with TurboID-OmpP2 proteins. (A) Venn diagram of TurboID-OmpP2-interacting host proteins and TurboID-interacting host proteins. (B) Molecular functions of host proteins interacting with the *G. parasuis* serotype 5 OmpP2 protein based on GO analysis. (C) KEGG analysis of host cell proteins interacting with the *G. parasuis* OmpP2 protein.

Affinity-purified proteins derived from His-TurboID-OmpP2-treated and His-TurboID-treated iPAM cells (in the presence of biotin) were subjected to mass spectrometric analysis. A total of 948 and 758 biotinylated host cell proteins interacting with His-TurboID-OmpP2 and His-TurboID were identified, respectively. Among them, 240 interacting proteins were unique in the His-TurboID-OmpP2 group (Fig. 2A). All 240 identified proteins were subjected to bioinformatics analysis. Three main types of annotations, biological processes, cellular components, and molecular functions, were obtained from the Gene Ontology (GO) consortium website. The biological process annotation showed that some proteins were involved in cellular processes, cellular component organization or biogenesis, metabolic processes, biological regulation, and response to stimulus. The cellular component annotation assigned other proteins to organelles, organelle parts, macromolecular complexes, membranes, and membrane parts. Enrichments based on the molecular function annotation were binding, catalytic activity, structural molecule activity, and transporter activity (Fig. 2B). Ten pathways, including gap junction, regulation of lipolysis in adipocytes, and renin secretion, were identified using the Kyoto Encyclopedia of Genes and Genomes (KEGG) reference pathway database to assign the 240 protein sequences (Fig. 2C). The purpose of our study was to identify host proteins involved in the recognition and phagocytosis of *G. parasuis* by iPAM cells. Membrane proteins are often involved in the recognition and phagocytosis of pathogens by iPAM cells, and so we were especially interested in membrane proteins. Of these 240 proteins, only four membrane proteins, CAV1, ARF6, PPP2R1A, and AP2M1, are membrane proteins, as identified by the UniProt website. The results of co-IP showed that AP2M1 could not interact with OmpP2 (see Fig. S1 in the supplemental material), so the functions of CAV1, ARF6, and PPP2R1A in the adhesion and phagocytosis of *G. parasuis* by iPAM cells were further studied. The mass spectrometry data are provided in Table S1.

### Further validation of interactions between *G. parasuis* OmpP2 protein and three host cell proteins.

**FIG 3:**
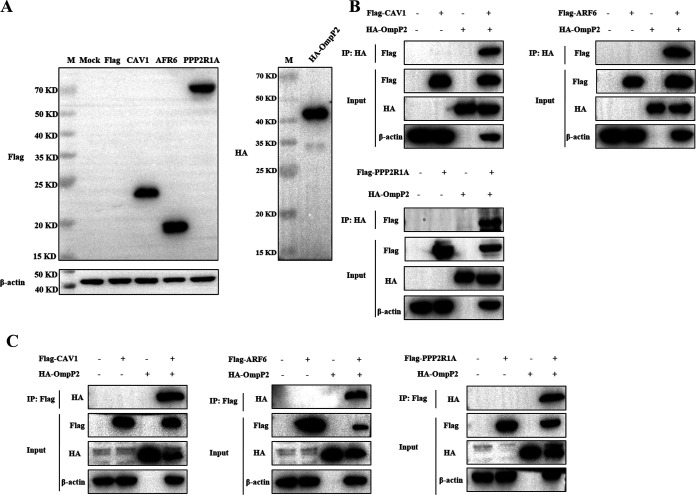
Validation of interactions between the OmpP2 protein and host cell proteins based on co-IP analysis. (A) Western blot analysis of the expression of Flag-CAV1, Flag-ARF6, and Flag-PPP2R1A plasmids transfected into HEK 293T cells and Pet-28a-HA-His-OmpP2 transformed into E. coli BL21(DE3). (B) Immunoblot of Flag-CAV1, Flag-ARF6, and Flag-PPP2R1A recombinant proteins from transfected HEK 293T cells and HA-OmpP2 protein using anti-HA MAb. (C) Immunoblot of HA-OmpP2 protein and host cell proteins precipitated using anti-Flag MAb from HEK 293T cells transfected with pCAGGS-Flag-CAV1, pCAGGS-Flag-ARF6, or pCAGGS-Flag-PPP2R1A.

Coimmunoprecipitation involving the three selected proteins (CAV1, ARF6, and PPP2R1A) was used to provide additional proof of the interaction between the host cell proteins and the *G. parasuis* serotype 5 OmpP2 protein. HEK 293T cells were transfected with expression plasmids to overexpress Flag-CAV1, Flag-ARF6, and Flag-PPP2R1A. The hemagglutinin (HA)-His-OmpP2 plasmid was transformed into E. coli BL21(DE3) to express the HA-His-OmpP2 fusion protein. The expression of these constructs was confirmed by Western blotting (Fig. 3A). In addition, co-IP was performed with anti-Flag or anti-HA monoclonal antibody (MAb) to capture protein complexes. As shown in Fig. 3, only OmpP2 and CAV1, ARF6, or PPP2R1A existed in the same reaction mixture, where corresponding bands could be observed in the IP samples (Fig. 3B and C). These results suggested that CAV1, ARF6, and PPP2R1A can interact with the OmpP2 protein. Therefore, we speculated that CAV1, ARF6, and PPP2R1A can affect the adhesion and phagocytosis of *G. parasuis* by iPAM cells.

### Overexpression of CAV1, ARF6, and PPP2R1A promoted adhesion and phagocytosis of *G. parasuis* in iPAM cells.

**FIG 4:**
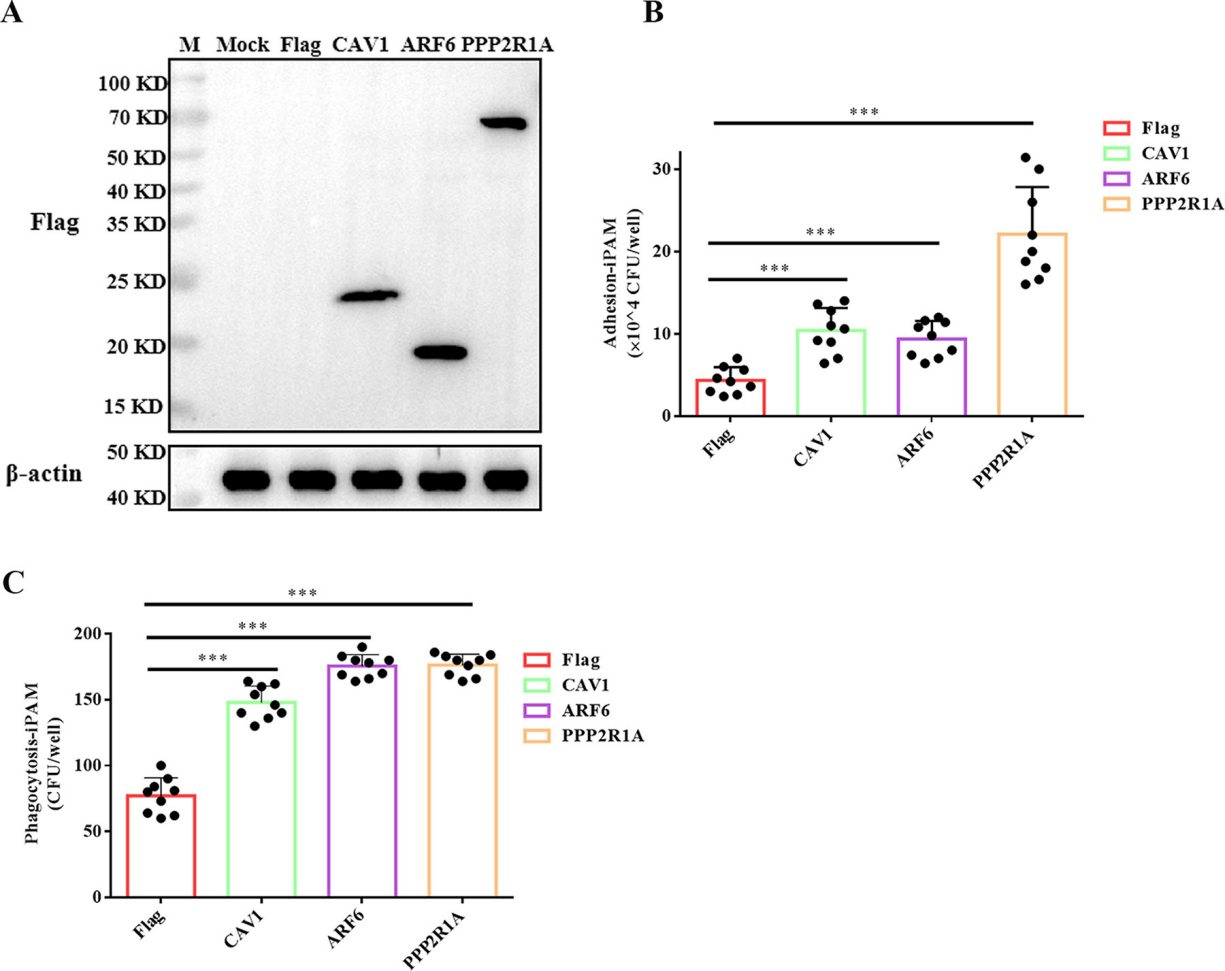
Overexpression of CAV1, ARF6, and PPP2R1A promoted the adhesion and phagocytosis of *G. parasuis* by iPAM cells. (A) Transfection of the expression plasmids and Western blot assessment of CAV1, ARF6, and PPP2R1A protein levels. (B) Ability of *G. parasuis* to adhere to transfected iPAM cells. Error bars represent SD of three independent experiments performed in triplicate. ***, *P < *0.001. (C) Phagocytosis of *G. parasuis* by transfected iPAM cells. Error bars represent SD of three independent experiments performed in triplicate. ***, *P < *0.001.

To investigate the potential role of CAV1, ARF6, and PPP2R1A in *G. parasuis* adherence and phagocytosis by iPAM cells, the overexpression efficiencies of CAV1, ARF6, and PPP2R1A were studied by Western blotting. Western blotting results indicated that all three proteins could be overexpressed (Fig. 4A). Furthermore, compared with the vector-transfected control group, the adherent and phagocytosed forms of *G. parasuis* both increased significantly in iPAM cells when CAV1, ARF6, or PPP2R1A was overexpressed (Fig. 4B and C). These results indicated that CAV1, ARF6, and PPP2R1A promoted the recognition and phagocytosis of *G. parasuis* by iPAM cells.

### Knockdown of CAV1, ARF6, and PPP2R1A gene expression inhibited adhesion and phagocytosis of *G. parasuis* by iPAM cells.

**FIG 5:**
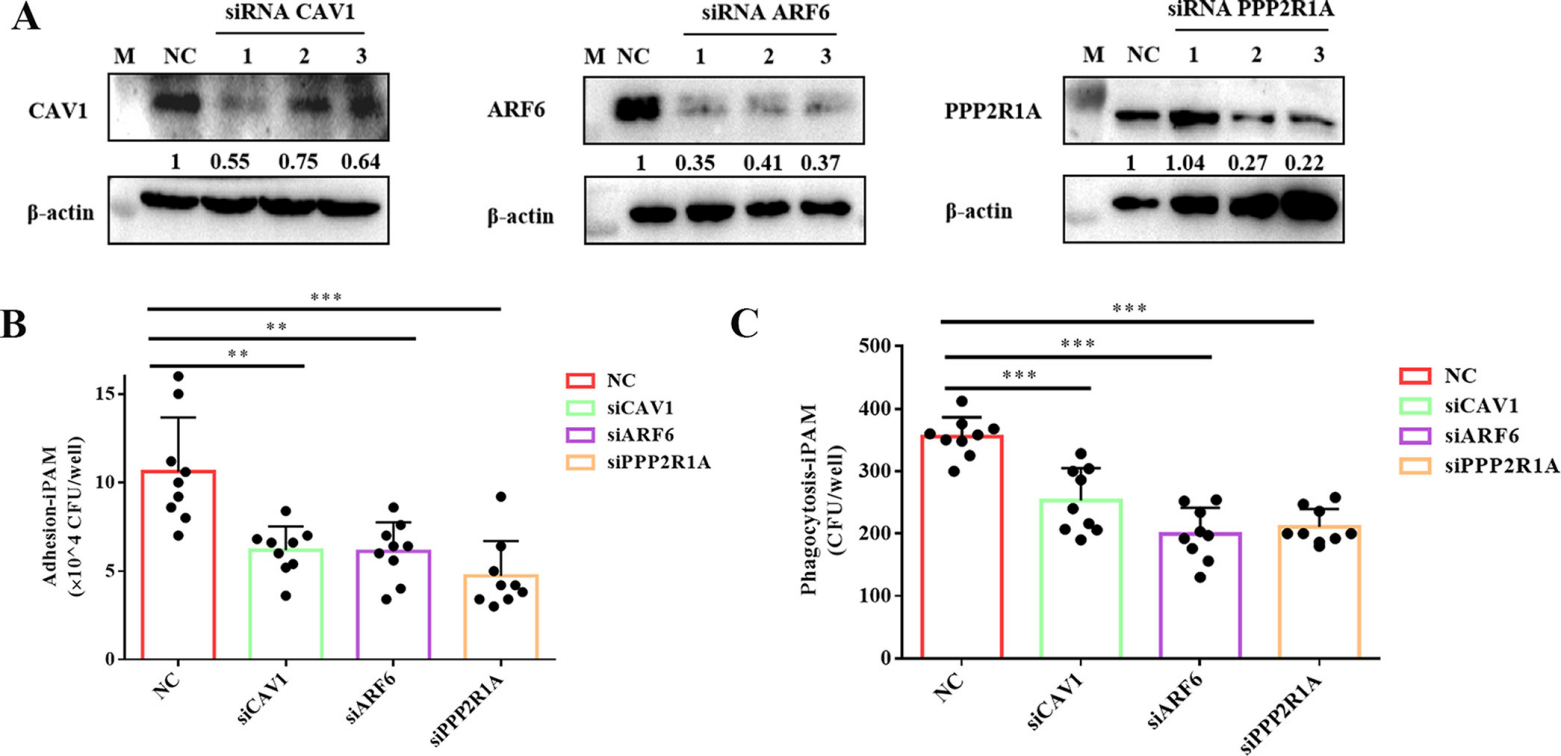
Knockdown of CAV1, ARF6, and PPP2R1A impairs the adhesion and phagocytosis of *G. parasuis* by iPAM cells. (A) siRNA silencing and Western blot assessment of CAV1, ARF6, and PPP2R1A protein levels. (B) Ability of *G. parasuis* to adhere to iPAM-silenced cells. Error bars represent SD of three independent experiments performed in triplicate. **, *P < *0.01; ***, *P < *0.001. (C) Phagocytosis ability of *G. parasuis* by iPAM-silenced cells. Error bars represent SD of three independent experiments performed in triplicate. ***, *P < *0.001.

To further confirm that CAV1, ARF6, and PPP2R1A are involved in the recognition and phagocytosis of *G. parasuis* by iPAM cells, a small interfering RNA (siRNA) assay was performed in iPAM cells. First, siRNA knockdown efficiencies against CAV1, ARF6, and PPP2R1A were detected by Western blotting. Western blotting results showed that most siRNAs were efficient in target gene knockdown, while siRNA CAV1 #1, siRNA ARF6 #1, and siRNA PPP2R1A #3 showed the best interference efficiencies (Fig. 5A). Therefore, siRNA CAV1 #1, siRNA ARF6 #1, and siRNA PPP2R1A #3 were selected for further study. The results of the adhesion and phagocytosis assays showed that, compared with the control group, both the adhesion and phagocytosis bacterial numbers of *G. parasuis* in iPAM cells decreased significantly when the cells were exposed to siCAV1, siARF6, or siPPP2R1A (Fig. 5B and C). These results further confirmed the involvement of CAV1, ARF6, and PPP2R1A in the recognition and phagocytosis of *G. parasuis* by iPAM cells.

### The OmpP2-CAV1, -ARF6, and -PPP2R1A interactions span the majority of its surface-exposed loops.

**FIG 6:**
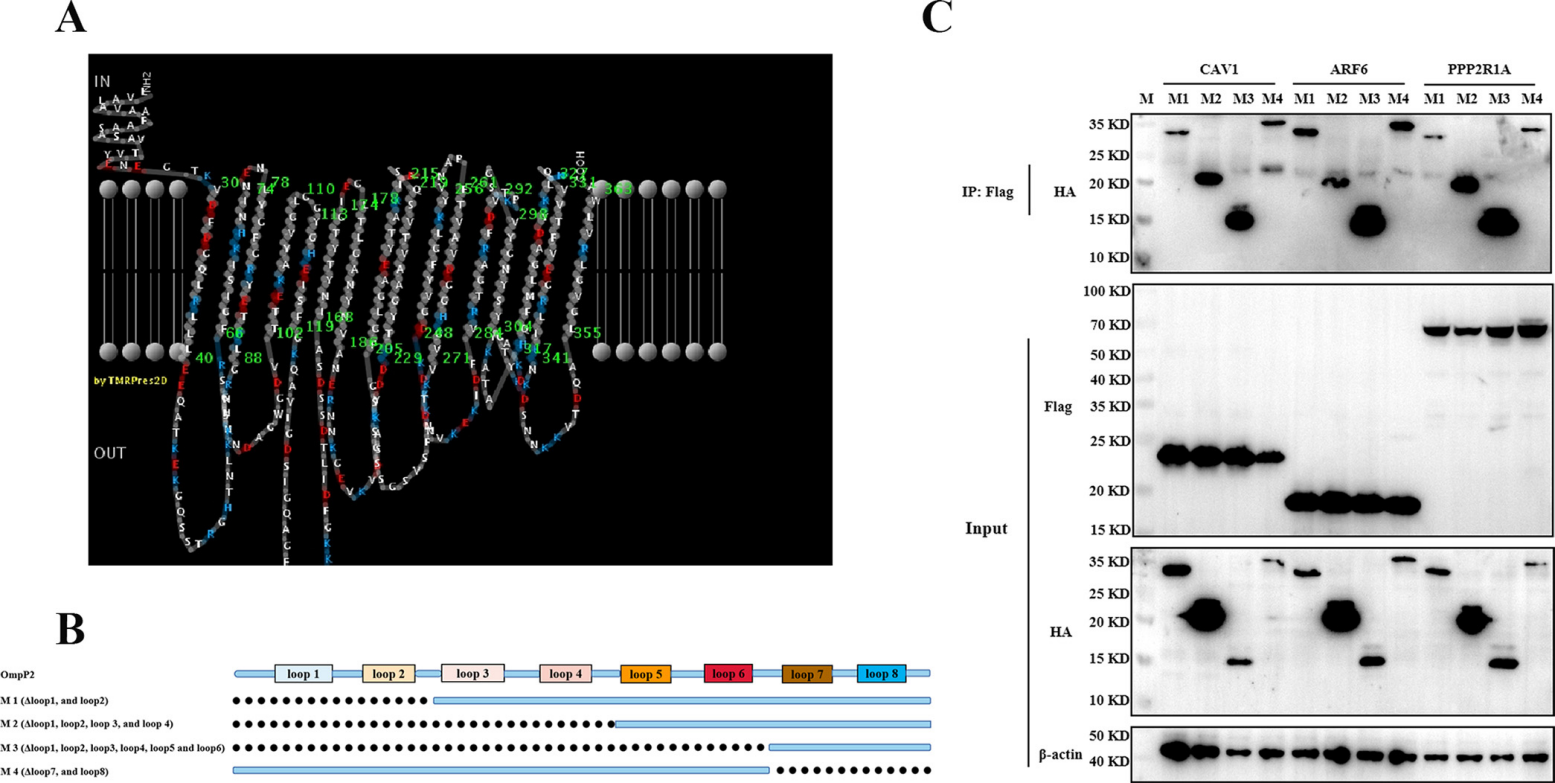
Several domains of OmpP2 are sufficient for CAV1, ARF6, and PPP2R1A interactions. (A) OmpP2 protein structural representation for the standard reference strain *G. parasuis* serotype 5. (B) Schematic of the OmpP2 domain architecture and constructs used in domain mapping studies. (C) Immunoblot using anti-Flag MAb for Flag-CAV1, Flag-ARF6, and Flag-PPP2R1A recombinant proteins from transfected HEK 293T cells and mutant proteins.

We confirmed that the OmpP2 protein of *G. parasuis* can interact with CAV1, ARF6, and PPP2R1A. In addition, we demonstrated that CAV1, ARF6, and PPP2R1A were involved in the recognition and phagocytosis of *G. parasuis* by iPAM cells. To further identify the key domain(s) of OmpP2 that interacted with CAV1, ARF6, and PPP2R1A, four truncated OmpP2 proteins were expressed and purified. The surface-exposed loops of OmpP2 were predicted using PRED-TMBB software (http://bioinformatics.biol.uoa.gr/PRED-TMBB/). As shown in Fig. 6A, OmpP2 of *G. parasuis* serotype 5 has eight surface-exposed loops. Based on this predicted structure, four truncated OmpP2 proteins, including M1 (with loop 1 and loop 2 deletions), M2 (with loops 1, 2, 3, and 4 deletions), M3 (with loops 1, 2, 3, 4, 5, and 6 deletions), and M4 (with loops 7 and 8 deletions), were expressed and purified (Fig. 6B) and further used in a co-IP assay to detect their interactions with host proteins. As shown in Fig. 6C, all the truncated proteins were observed to interact with CAV1, ARF6, and PPP2R1A (Fig. 6C), suggesting that the region of OmpP2 interaction spans the majority of the OmpP2 surface-exposed loops. This means that several surface-exposed loops of OmpP2 are cooperatively involved in the interactions with host proteins.

## DISCUSSION

*Glaesserella parasuis* is a common pathogen present in the upper respiratory tract of pigs. *G. parasuis* phagocytosis by alveolar macrophages may lead to intracellular killing (5, 21). However, virulent *G. parasuis* can resist phagocytosis by alveolar macrophages (22). In the lung, the first line of defense is alveolar macrophages, whose main role is to eliminate pathogenic microorganisms and other environmental particles (23–25). *G. parasuis* must avoid phagocytosis by alveolar macrophages to survive *in vivo* and induce disease. In contrast, macrophages initiate the phagocytosis process after their surface membrane proteins bind to and recognize microbes. However, little is known about the surface membrane proteins of alveolar macrophages involved in the recognition and phagocytosis of *G. parasuis*. In this study, we applied a TurboID proximity labeling method to identify interacting cellular proteins with OmpP2 of *G. parasuis*, and we identified 240 unique interacting host proteins with OmpP2 of *G. parasuis* serotype 5, of which three membrane proteins (CAV1, ARF6, and PPP2R1A) were confirmed to be involved in the recognition and phagocytosis of *G. parasuis* by iPAM cells.

Caveolins (CAVs) are a family of membrane proteins involved in the formation of the caveola and receptor-dependent endocytosis. CAV1 and CAV2 are coexpressed in various cells, such as airway epithelial cells, endothelial cells, and type I lung cells. CAV1 is the main component of flask-shaped plasma membrane invagination and caveolae, which play essential roles in host defense against infections (26, 27). A previous study found that CAV1 promoted the phagocytosis of pulmonary epithelial cells against Pseudomonas aeruginosa by lipid raft-mediated endocytosis (27). In addition, CAV1^−/−^ mice displayed decreased phagocytosis ability and higher bacterial burdens in Klebsiella pneumoniae infection (28). In this study, we found that overexpressing CAV1 in iPAM cells significantly promoted the recognition and phagocytosis of *G. parasuis* by iPAM cells. In contrast, the ability of iPAM cells to phagocytose *G. parasuis* was significantly reduced after CAV1 knockdown. These results suggested that CAV1 is involved in the recognition and phagocytosis of *G. parasuis* by iPAM cells.

ADP ribosylation factors (ARFs) are small GTP-binding proteins that play crucial roles in lipid metabolism and signaling transduction, membrane trafficking, and actin cytoskeleton remodeling (29–31). Six ARF proteins are present in mammals and are grouped into three classes based on their structural similarity: class I (ARF1, ARF2, and ARF3), class II (ARF4 and ARF5), and class III (ARF6). ARF6 is the only member of class III that localizes to the endocytic system and the mammalian plasma membrane. ARF6 is involved in different biological processes, including cytokinesis, endocytosis, and the organization of the actin cytoskeleton (30). ARF6 is involved in the invasion of host cells by Salmonella enterica serovar Typhimurium, which facilitates the establishment of intracellular infection (32). In this research, when ARF6 was overexpressed in cells, the ability of iPAM cells to recognize and phagocytose *G. parasuis* was significantly enhanced. When this gene was knocked down by siRNA, the capacity of iPAM cells to phagocytose *G. parasuis* was significantly reduced. The results of this study were consistent with those of previous studies showing that ARF6 is also involved in the recognition and phagocytosis of *G. parasuis* by iPAM cells.

PP2A, one of the four major serine/threonine phosphatases, is a heterotrimeric phosphatase consisting of a catalytic subunit (PP2Ac), a B regulatory subunit, and a scaffold subunit (PR65) (33, 34). PP2A regulates multiple cellular functions through a broad spectrum of substrates, including mitosis, DNA damage repair, and cell cycle regulation (33, 35–37). PPP2R1A is one of the isoforms of PR65, and another isoform is PPP2R1B. At present, most studies have shown that PPP2R1A is associated with the occurrence of cancer and can bind to the T antigen of polyomavirus and simian virus 40 (33). In human melanoma, lung cancer, and breast cancer, somatic mutations (an R-to-W change at position 418 [R418W], E64D, and E64G) in PPP2R1A have been identified (38). These mutations promote human cell transformation by disrupting the composition of the PP2A complex and reducing phosphatase activity (39). Until now, PPP2R1A has not been reported to be involved in the process of pathogen-host interaction. In the present study, we found that iPAM cells significantly enhanced the recognition and phagocytosis of *G. parasuis* when PPP2R1A was overexpressed. Meanwhile, when the PPP2R1A gene was silenced by siRNA, the phagocytic ability of iPAM cells to *G. parasuis* was obviously reduced. This suggested that PPP2R1A is involved in the recognition and phagocytosis of pathogens by host cells.

Mayank Srivastava et al. identified neural cell adhesion molecule 1 as a potential receptor of Zika virus by a chemical labeling method (40). However, this method requires synthesizing a chemical probe to label viruses, and the probe is very difficult to design and synthesize, limiting its applications. Our study provides new insight into host-pathogenic microorganism interactions. The TurboID proximity labeling system could be applied to study the interactions of zoonotic agents such as Salmonella, Brucella, and Streptococcus with host proteins to find the potential receptors or key proteins that cause infection. These findings will lay a theoretical foundation for preventing, controlling, and treating disease, breeding disease-resistant animals, and developing vaccines.

In summary, we identified 240 unique interacting proteins in the His-TurboID-OmpP2 group. Among them, only four membrane proteins, CAV1, ARF6, PPP2R1A, and AP2M1, were identified. CAV1, ARF6, and PPP2R1A were further proven to directly interact with OmpP2 of *G. parasuis* by co-IP assay. Finally, we found that CAV1, ARF6, and PPP2R1A were involved in the recognition and phagocytosis of *G. parasuis* by iPAM cells. This study first revealed the interacting proteome of iPAM cells with the *G. parasuis* serotype 5 OmpP2 protein by the TurboID proximity labeling system and confirmed that CAV1, ARF6, and PPP2R1A are involved in the recognition and phagocytosis of *G. parasuis* by iPAM cells. However, the recognition and phagocytosis mechanism of *G. parasuis* by iPAM cells requires in-depth research. These results provide new insight into a better understanding of Glasser’s disease pathogenesis.

## MATERIALS AND METHODS

### Bacterial strains and culture conditions.

The standard reference strain of *G. parasuis* serotype 5 was preserved in our laboratory. *G. parasuis* was cultured in tryptic soy broth (TSB) medium or tryptic soy agar (TSA) plates supplemented with 10 μg/mL NAD and 8% inactivated cattle serum at 37°C. Escherichia coli DH5α and Escherichia coli BL21(DE3) were cultured in TSA plates or Luria-Bertani medium at 37°C.

### Cells and culture conditions.

The immortalized porcine alveolar macrophage (iPAM) cell line, provided by XueHui Cai, Harbin Veterinary Research Institute of Chinese Academy of Agricultural Sciences (41), was cultured and maintained in RPMI 1640 medium with 10% fetal bovine serum (FBS). The human embryonic kidney 293T (HEK 293T) cell line was obtained from the American Type Culture Collection and cultured and maintained in Dulbecco’s modified Eagle’s medium with 10% FBS.

### Creation of plasmids.

The pcDNA-TurboID plasmid was preserved in our laboratory. To generate the His-TurboID-OmpP2 fusion construct, TurboID and OmpP2 fragments were amplified by PCR (Phanta Super-Fidelity DNA polymerase [Vazyme, China]) using pcDNA-TurboID and the *G. parasuis* serotype 5 genome as templates, respectively. These two fragments were linked with overlap extension PCR to construct a new fragment, TurboID-OmpP2, which was then inserted into the pET-28a plasmid with NheI and XhoI restriction enzymes to generate the recombinant expression plasmid pET-28a-TurboID-OmpP2. His-TurboID was amplified using primers P5 and P6 from pcDNA-TurboID and cloned into the pET-28a plasmid with NheI and XhoI restriction enzymes to generate the recombinant expression plasmid pET-28a-TurboID. The two recombinant plasmids were confirmed by sequencing.

The pET-28a-HA-His plasmid, which can express both HA tag and His tag, was preserved in our laboratory. HA-OmpP2 was amplified using primers P7 and P8 from the *G. parasuis* serotype 5 genome and cloned into the pET-28a-HA-His plasmid with NheI and XhoI restriction enzymes to generate plasmid pET-28a-HA-His-OmpP2. Four truncated OmpP2 protein expression constructs, pET-28a-HA-His-OmpP2-M1, pET-28a-HA-His-OmpP2-M2, pET-28a-HA-His-OmpP2-M3, and pET-28a-HA-His-OmpP2-M4, were generated from pET-28a-HA-His-OmpP2 using primer pairs P9-P10, P11-P10, P12-P10, and P13-P14, respectively. The quality of the plasmids was confirmed by sequencing.

The coding DNA sequences of CAV1 (caveolin 1), ARF6 (ADP ribosylation factor 6), and PPP2R1A (protein phosphatase 2 scaffold subunit A alpha) were amplified from iPAM cells by reverse transcription-PCR using primer pairs P15-P16, P17-P18, and P19-P20, respectively, and cloned into the pCAGGS-Flag vector with EcoRI and XhoI, EcoRI and XhoI, EcoRI and NheI restriction enzymes to generate the recombinant expression plasmids Flag-CAV1, Flag-ARF6, and Flag-PPP2R1A, respectively. All the plasmids were verified by sequencing. The primers used in this study are listed in Table 1.

**TABLE 1:** Primers used for plasmid construction

Primer	Sequence	Purpose
P1	CTAGCTAGCGGTGGCAGCGGTGGCAGCATGAAAGACAATACTGTGCC	To amplify the TurboID fragment
P2	GCTACTAGTGTTTTTTTCATGCTGCCACCGCTGCCACCCTTTTCGGCAGACCGCAGAC	
P3	GTCTGCGGTCTGCCGAAAAGGGTGGCAGCGGTGGCAGCATGAAAAAAACACTAGTAGC	To amplify the OmpP2 fragment
P4	CCGCTCGAGCCATAATACACGTAAACCAAC	
P5	CTAGCTAGCGGTGGCAGCGGTGGCAGCATGAAAGACAATACTGTGCC	To amplify the His-TurboID-fragment
P6	CCGCTCGAGCTTTTCGGCAGACCGCAGACTG	
P7	GCGGCAGCCATATGGCTAGCGGTGGCAGCGGTGGCAGCATGAAAAAAACACTAGTAGC	To amplify the HA-His-OmpP2 fragment
P8	CCGCTCGAGCCATAATACACGTAAACCAAC	
P9	GGAATTCCATATGGGTGGCAGCGGTGGCAGCATGGGTGGCTATGGTCATGAAAT	To generate pET-28a-HA-His-OmpP2-M1
P10	CCGCTCGAGCCATAATACACGTAAACCAAC	
P11	GGAATTCCATATGGGTGGCAGCGGTGGCAGCATGGCGGAAAGTCAATCTGTA	To generate pET-28a-HA-His-OmpP2-M2
P12	GGAATTCCATATGGGTGGCAGCGGTGGCAGCATGACTCCAAAATCTGGCGTGTATG	To generate pET-28a-HA-His-OmpP2-M3
P13	GGAATTCCATATGGGTGGCAGCGGTGGCAGCATGAAAAAAACACTAGTAGC	To generate pET-28a-HA-His-OmpP2-M4
P14	CCGCTCGAGAACATCAAATCTTGCGCCAG	
P15	CCGGAATTCGGTGGCAGCGGTGGCAGCATGTCGGGGGGCAAATACGTAG	To amplify CAV1 gene
P16	CCGCTCGAGTTATATTTCTTTCTGCATG	
P17	CCGGAATTCGGTGGCAGCGGTGGCAGCATGGGGAAGGTGCTATCTAAG	To amplify ARF6 gene
P18	CCGCTCGAGTTAGGATTTGTAGTTAGAGG	
P19	CCGGAATTCGGTGGCAGCGGTGGCAGCATGGCGGCGGCCGACGGCGATG	To amplify PPP2R1A gene
P20	CTAGCTAGCTCAGGCGAGCGACAGAACAG	

### Expression of His-TurboID-OmpP2, His-TurboID, HA-His-OmpP2, and truncated proteins.

The plasmids pET-28a-TurboID-OmpP2, pET-28a-TurboID, pET-28a-HA-His-OmpP2, pET-28a-HA-His-OmpP2-M1, pET-28a-HA-His-OmpP2-M2, pET-28a-HA-His-OmpP2-M3, and pET-28a-HA-His-OmpP2-M4 were transformed into E. coli BL21(DE3) for recombinant protein expression. Transformants were cultured in LB medium containing 50 μg/mL kanamycin at 37°C for 16 h, transferred into fresh LB medium containing 50 μg/mL kanamycin at a ratio of 1:10, and allowed to grow at 37°C with shaking until the optical density at 600 nm reached ~0.6. Isopropyl-β-d-thiogalactopyranoside (IPTG; 0.5 mM) was then added to the cultures to induce recombinant protein expression.

The IPTG-induced bacteria were pelleted at 8,000 rpm for 20 min at 4°C and resuspended in ice-cold resuspension buffer (1 M Tris-HCl [pH 7.4], 0.5 M NaCl, 20 mM imidazole, protease inhibitor cocktail) and disrupted three times under 25,000 lb/in^2^ by using a French press (constant system) at 4°C. Crude extracts were collected by centrifugation at 12,000 rpm for 15 min at 4°C. After centrifugation, the supernatant was filtered through a 0.22-μm membrane to remove any debris.

The obtained supernatant was loaded on a HisSep Ni-nitrilotriacetic acid agarose resin column (2 mL; Yeasen, Shanghai, China) preequilibrated with 10 column volumes of ice-cold buffer A (1 M Tris-HCl, 0.5 M NaCl; pH 7.4). Filtrated crude extracts were subsequently loaded onto the column at a flow rate of 0.5 mL/min at 4°C, and the protein-bound resin was washed with phosphate-buffered saline (PBS) (pH 7.4) containing 20 mM imidazole at a flow rate of 1 mL/min. Buffer B in this step contained the same buffering species but a higher imidazole concentration (0.5 M imidazole). After the sample was loaded onto the column, proteins were eluted by a 15-min linear gradient elution (the buffer composition was changed from 0% of buffer B to 100% of buffer B within 15 min). SDS-PAGE and Coomassie blue staining were then used to detect the purity of each collected fraction. The fractions that contained pure recombinant proteins were pooled accordingly and subsequently concentrated.

### Biotin and streptavidin affinity purification.

The iPAM cells were seeded into 6-well tissue culture plates and cultured in RPMI 1640 medium containing 10% FBS for 24 h. After the cells had grown to 100% confluence, they were washed three times with sterile PBS, and 16 μg His-TurboID-OmpP2 or His-TurboID recombinant protein was added to fresh RPMI 1640 medium and incubated on ice for 1 h to allow the proteins to be recognized by iPAM cells. Then, 50 μM biotin (Sigma) was added and incubated at 37°C for 20 min to label the interacting proteins. The reaction was then quenched, and the cells were washed three times by removing the suspension liquid and replacing it with ice-cold PBS. Cells (two wells per sample) were then lysed in 500 μL lysis buffer (cell lysis buffer for Western and IP [Beyotime, China]) containing a protease inhibitor cocktail. Residual cells and cell debris adhering to the culture plates were scraped into the lysates, and after incubation on an end-over-end rotator at 4°C for 1 h, they were centrifuged at 14,000 × *g* for 10 min at 4°C. Supernatants were incubated with streptavidin magnetic beads (NEB, USA) on a rotator at 4°C overnight (250 μL beads per sample) that were previously washed with binding buffer (20 mM Tris-HCl [pH 7.5], 0.5 M NaCl, 1 mM EDTA). Then, the beads were washed three times with cell lysis buffer. Biotinylated proteins were then eluted by boiling the beads in 100 μL SDT buffer (4% [wt/vol] SDS, 100 mM Tris-HCl, 1 mM dithiothreitol; pH 7.6), followed by mass spectrometry or SDS-PAGE.

### Mass spectrometry analysis.

The samples were analyzed by mass spectrometry in the Omics Space (Shanghai, China). The acquired data from triplicate MS runs for each sample were combined and searched against an UniProt_Sus_scrofa_333904_20190905.fasta protein sequence database using the MaxQuant computational proteomics platform version 2.0.1.0. Proteins were identified using the Andromeda peptide search engine integrated into the MaxQuant environment. A decoy version of the self-database was used to estimate the peptide and protein false-discovery rates. The maximum protein and peptide-spectrum match (PSM) false-discovery rates were set to 0.01. Carbamidomethylation of cysteine was set as a fixed modification, with protein oxidation of methionine set as a variable modification (enzyme: trypsin/P; maximum number of missed cleavages: 2). We used MaxQuant to determine the label-free quantification (LFQ), a measure of protein abundance. The LFQ value was obtained by dividing protein intensities by the number of theoretically observable tryptic peptides between 5 and 30 amino acids and was on average highly correlated with protein abundance. The Retrieve/ID Mapping tool was utilized at www.UniProt.org for subcellular location designations of identified candidate proteins.

### Overexpression and RNA interference experiments.

For the overexpression assay, 5 × 10^5^ iPAM cells were seeded into 24-well tissue culture plates in RPMI 1640 medium containing 10% FBS. At 60% to 70% confluence, cells were transfected with Flag-CAV1, Flag-ARF6, or Flag-PPP2R1A expression plasmids and Flag-vector, respectively, using jetPRIME transfection reagent (Polyplus) according to the manufacturer’s instructions for 24 h. For siRNA-mediated knockdown, 5 × 10^5^ iPAM cells were seeded into 24-well tissue culture plates in RPMI 1640 medium containing 10% FBS. After the cells had grown to 60% to 70% confluence, they were transfected with 50 nM CAV1-targeting, ARF6-targeting, or PPP2R1A-targeting siRNA or negative-control siRNA using jetPRIME transfection reagent as recommended by the manufacturer for 24 h. Double-stranded siRNAs targeting CAV1, ARF6, and PPP2R1A and a negative control were designed and synthesized by GenePharma Co. (Shanghai, China) (Table 2).

**TABLE 2:** siRNA sequences used in this study

Gene	No.	Orientation	siRNA sequence (5′–3′)
CAV1	1	Sense	GGAAAUGAACGAGAAGCAATT
		Antisense	UUGCUUCUCGUUCAUUUCCTT
	2	Sense	CCGUUGUACCCUGCAUUAATT
		Antisense	UUAAUGCAGGGUACAACGGTT
	3	Sense	GCAAUAUCCGCAUCAACAUTT
		Antisense	AUGUUGAUGCGGAUAUUGCTT
ARF6	1	Sense	GCAAGACAACCAUCCUGUATT
		Antisense	UACAGGAUGGUUGUCUUGCTT
	2	Sense	GCUACACCGCAUUAUCAAUTT
		Antisense	AUUGAUAAUGCGGUGUAGCTT
	3	Sense	GCUCACAUGGUUAACCUCUTT
		Antisense	AGAGGUUAACCAUGUGAGCTT
PPP2R1A	1	Sense	GCAACGAGGAUGUUCAGCUTT
		Antisense	AGCUGAACAUCCUCGUUGCTT
	2	Sense	GGAGAAUGUCAUCAUGACUTT
		Antisense	AGUCAUGAUGACAUUCUCCTT
	3	Sense	GGUCAAGCCCAUCCUAGAGTT
		Antisense	CUCUAGGAUGGGCUUGACCTT
Negative control		Sense	UUCUCCGAACGUGUCACGUTT
		Antisense	ACGUGACACGUUCGGAGAATT

### Coimmunoprecipitation assay.

To analyze the interaction between OmpP2 and host interactors, HEK 293T cells seeded into 6-well culture plates were transfected with the corresponding expression plasmids. Transfected cells were harvested at 48 h posttransfection and lysed in cell lysis buffer containing 1 mM protease inhibitor. After centrifugation at 14,000 × *g* for 10 min, the lysate supernatants containing 1 to 2 mg of total protein were incubated with 2.5 μg HA-OmpP2 or truncated OmpP2 proteins for 4 h with gentle rocking at 4°C and then incubated overnight with mouse MAb against Flag or HA tag with gentle rocking at 4°C. Protein A/G beads washed with cell lysate were added to the supernatants and incubated with gentle rocking for 4 h at 4°C. The beads were washed four times with cold cell lysate and boiled with 1× SDS loading buffer for 10 min, followed by Western blotting.

### Western blotting.

The cells were collected, and cell proteins were extracted using a total protein extraction kit (Beyotime, China) according to the manufacturer’s instructions. The proteins were isolated by SDS–12% PAGE and then electrophoretically transferred onto polyvinyl difluoride (PVDF) membranes (Millipore). The PVDF membranes were blocked with 5% bovine serum albumin at room temperature for 2 h and then incubated with the respective primary antibodies overnight at 4°C. After washing three times with Tris-buffered saline with Tween 20, the membranes were incubated with HRP-linked goat anti-rabbit or HRP-linked goat anti-mouse antibody at room temperature for 2 h and visualized using an Omni-ECL Femto light chemiluminescence kit (Epizyme Biotech, China). The antibodies used in this study are listed in Table 3.

**TABLE 3:** Antibodies used in this study

Antibody	Name	Supplier	Catalog no.
CAV1	Caveolin-1 rabbit MAb	Beyotime	AF1231
ARF6	ARF6 rabbit polyclonal Ab	Abbkine	ABP55026
PPP2R1A	PPP2R1A rabbit polyclonal Ab	Proteintech	15882-1-AP
β-Actin	β-Actin rabbit MAb(high dilution)	ABclonal	AC026
Flag	DYKDDDDK tag mouse MAb	Proteintech	66008-4-Ig
HA	HA tag mouse MAb	Proteintech	66006-2-Ig
Secondary antibodies^*a*^			
	HRP goat anti-mouse IgG (H+L)	ABclonal	AS003
	HRP goat anti-rabbit IgG (H+L)	ABclonal	AS014
	HRP-conjugated streptavidin	Sigma	RABHRP3
	HRP-conjugated 6×His-tagged MAb	Proteintech	HRP-66005
	HRP goat anti-mouse IgG HCS	Abbkine	A25112
	HRP goat anti-mouse IgG LCS	Abbkine	A25012

a H, heavy chain; L, light chain; HCS, heavy chains; LCS, light chains.

### Adhesion and phagocytosis assays.

The adhesion and phagocytosis assays were performed based on previously described methods with some minor modifications (4, 24). For the adhesion assay, the iPAM cells were subjected to overexpression or siRNA as described above, washed thrice with sterile PBS, and infected with approximately 1 × 10^7^ CFU *G. parasuis*. The culture plates were thereafter incubated at 37°C for 2 h to allow bacterial adhesion. The plates were then washed five times with sterile PBS to eliminate nonspecific bacterial attachment and then incubated with 200 μL 0.25% trypsin–EDTA at 37°C for 10 min. After incubation, the cells were resuspended from the bottom of every well. The cell suspensions with adherent bacteria were diluted 10-fold and placed onto TSA plates supplemented with NAD and serum at 37°C for 24 h, and the number of bacteria was recorded. For the phagocytosis assay, cell treatment, bacterial infection, and counting of bacterial colonies were performed as described above for the bacterial adherence assay except that the extracellular bacteria were eliminated by incubation of RPMI 1640 medium containing 100 U/mL penicillin and 10 μg/mL streptomycin sulfate for another 1 h to facilitate the killing of extracellular bacteria. The cells were then washed and lysed as described above. All the above-described assays were performed in triplicate and repeated three times independently.

### Statistical analysis.

The results are presented as the means ± standard deviations (SD). The results were evaluated by a multiple *t* test in GraphPad Prism 7.0 (GraphPad Software Inc., USA). A *P* value (*) of <0.05 was considered statistically significant, while *P* values of <0.01 (**) and <0.001 (***) were regarded as highly significant.
